# Estimating PM_2.5_ Exposures and Cardiovascular Disease Risks in the Yangtze River Delta Region Using a Spatiotemporal Convolutional Approach to Fill Gaps in Satellite Data

**DOI:** 10.3390/toxics13050392

**Published:** 2025-05-14

**Authors:** Muhammad Jawad Hussain, Myeongsu Seong, Behjat Shahid, Heming Bai

**Affiliations:** 1 Research Center for Intelligent Information Technology, Nantong University, Nantong 226019, China; 2 Department of Mechatronics and Robotics, School of Advanced Technology, Xi’an Jiaotong-Liverpool University, Suzhou 215123, China; myeongsu.seong@xjtlu.edu.cn; 3 Faculty of Media and Communication Studies, University of Central Punjab, Lahore 54590, Pakistan; behjatshahid@gmail.com

**Keywords:** PM_2.5_, aerosol optical depth, top of atmospheric reflectance, cardiovascular diseases, machine-learning

## Abstract

Accurate estimation of ambient PM_2.5_ concentrations is crucial for assessing air quality and health risks, particularly in regions with limited ground-based monitoring. Satellite-retrieved data products, such as top-of-atmosphere reflectance (TOAR) and aerosol optical depth (AOD), are widely used for PM_2.5_ estimation. However, complex atmospheric conditions cause retrieval gaps in TOAR and AOD products, limiting their reliability. This study introduced a spatiotemporal convolutional approach to fill sampling gaps in TOAR and AOD data from the Himawari-8 geostationary satellite over the Yangtze River Delta (YRD) in 2016. Four machine-learning models (random forest, extreme gradient boosting, gradient boosting, and support vector regression) were used to estimate hourly PM_2.5_ concentrations by integrating gap-filled and original TOAR and AOD data with meteorological variables. The random forest model trained on gap-filled TOAR data yielded the highest predictive accuracy (R^2^ = 0.75, RMSE = 18.30 μg m^−3^). Significant seasonal variations in PM_2.5_ estimates were found, with TOAR-based models outperforming AOD-based models. Furthermore, we observed that a substantial portion of the YRD population in non-attainment areas is at risk of cardiovascular disease due to chronic PM_2.5_ exposure. This study suggests that TOAR-based models offer more reliable PM_2.5_ estimates, enhancing air-quality assessments and public health-risk evaluations.

## 1. Introduction

Environmental pollution is one of the leading causes of disease and premature deaths worldwide [1,2,3] and often results from rapid unplanned urbanization, industrialization, economic development, and increased motorization over recent decades [4,5]. Among the most harmful atmospheric pollutants is ambient fine particulate matter (PM_2.5_) which is characterized by an aerodynamic diameter of less than 2.5 μm. It significantly impacts the environment, the climate [6], and human health [7,8].

PM_2.5_ particles can consist of liquid droplets, dry solid fragments, or a combination of both, such as solid cores coated with liquids. Particles that consist fully or partially of liquid droplets are known as aerosols [9]. These pollutants originate from a variety of sources, including volcanic ash, wildfires, and industrial emissions [3]. However, many of these particles are produced when chemical compounds released by human activities, such as factory and auto emissions, coal combustion, and biomass burning, combine in the atmosphere [9,10]. These particles may contain various substances, including nitrate, ammonium, metallic compounds, organic carbonaceous material, sulfate, and elemental carbon [11].

In the Yangtze River Delta (YRD) region, the physicochemical properties of PM_2.5_ vary significantly due to seasonal changes, geographical features, and local emission factors. During winter, high concentrations of carbonaceous aerosols, such as black carbon and organic carbon, are prevalent due to heating and coal combustion, while sulfates and nitrates dominate from industrial emissions and vehicle exhaust. In contrast, summer sees an increase in secondary organic aerosols due to photochemical reactions, along with sea salt from coastal areas. The region’s humid subtropical climate, mountainous topography, and land–sea contrasts complicate pollutant dispersion, leading to high PM_2.5_ concentrations, especially in urban and rural areas.

The chemical composition of PM_2.5_ in the YRD includes organic carbon, black carbon, sulfates, nitrates, and ammonium salts, all of which contribute to health risks, including risks of respiratory diseases and cardiovascular problems. Due to the existing evidence for the toxic effects of these compounds on human health, they pose a growing concern for global health systems [12,13]. In particular, due to the tiny size of PM_2.5_ pollutant particles, they can bypass the natural defense mechanism of the human respiratory system and enter the human body, affecting the lungs and cardiovascular systems [14]. Exposure to PM_2.5_ has been linked to a variety of health issues, including cardiovascular diseases, stroke, lung cancer, and respiratory infections [15,16,17,18,19,20]. The impact of PM_2.5_ exposure on cardiovascular diseases is significant because these diseases are among the major causes of death in most countries [21,22]. Specifically, in China, where a large portion of the population is middle-aged and exposed to PM_2.5_ concentrations that exceed recommended standards, cardiovascular and respiratory diseases account for the greatest proportion of the disease burden [23,24,25,26]. Therefore, accurate estimates of ground-level PM_2.5_ concentration are essential for effectively addressing the major risk factors for cardiovascular disease, many other health issues, and environmental challenges [27,28,29]. Early detection and prevention of PM_2.5_ exposure can help reduce its harmful impact on human health and lower the risks of cardiovascular disease and other associated diseases [3,22].

Currently, the primary methods for determining PM_2.5_ concentrations are ground-based station measurements, satellite remote-sensing retrievals, and numerical model simulations [30]. In 2012, China built its national-scale PM_2.5_-concentration ground monitoring network. However, more than 90% of the monitoring locations are in urban areas [31]. As a result, when the health impacts of PM_2.5_ exposure are examined over a greater geographic region, the disproportionate contribution of the urban-based observations may bring bias into the final results [32]. As an alternative, satellite data have been increasingly important for providing continuous geographic and temporal coverage of PM_2.5_ concentrations in recent years. As a result, various techniques, including statistical and machine-learning models, have been developed to estimate PM_2.5_ using satellite data products [33,34,35].

At present, satellite aerosol optical depth (AOD) data products are usually utilized to estimate PM_2.5_ concentrations over continuous temporal and spatial coverage. The low spatial resolution and significant spatial gaps in the AOD data products reduce their effectiveness and broader applicability [36]. However, the spatial resolution of AOD products has improved over time; for example, the resolution improved from 10 km (as in MOD04) to 1 km (as in MAIAC). As a result, the methodology for estimating surface PM_2.5_ from AOD has matured over time, with improvements in the accuracy of retrieval techniques and spatial resolution of AOD products [37]. Nevertheless, despite improvements in current aerosol-retrieval algorithms and high spatial resolution, AOD products still suffer from various limitations. The most significant limitation might be the problems of missing data (spatial and temporal gaps), which are caused by several factors, including the sensor’s limited capability, particularly over high-altitude regions; unfavorable environmental conditions created by cloud contamination; extremely high aerosol concentrations (such as those caused by wildfires or volcanic ash); bright surfaces (desert or snow); and several other retrieval challenges [38,39,40,41]. As a result, the AOD–PM_2.5_ relationship can be difficult to determine in grid cells having missing or abnormal values, especially for daily PM_2.5_ assessments. The result is the limited coverage of the AOD-based PM_2.5_-retrieval methods, especially on days with high levels of pollution and in highly polluted regions [42]. When one is examining the effects of PM_2.5_ on human health and the environment, however, a gapless remotely sensed high-resolution AOD dataset is especially important [43]. The majority of existing gap-filling techniques for AOD data typically fill daily data gaps by utilizing statistical methodologies or spatially interpolating neighboring PM_2.5_ values [38,40,44,45]. These methods perform poorly when the data cover large geographic regions with few monitoring stations.

Another effective approach to addressing the aforementioned challenges is replacing AOD products with top-of-atmosphere reflectance (TOAR) products to estimate PM_2.5_ concentrations [46]. TOAR data products provide a higher spatial resolution and broader coverage than AOD products and contain signals from both the surface and the atmosphere that can be used to derive information about atmospheric pollution [47,48]. Therefore, many previous studies have tried to evaluate the feasibility of this approach and utilized satellite TOAR data products to estimate PM_2.5_ concentrations with greater spatial resolution and coverage by employing machine-learning [36,46,49] and deep-learning techniques [47] as retrieval models. These studies demonstrated the feasibility of directly acquiring PM_2.5_ concentrations from satellite TOAR data. However, these studies still have some limitations. For example, TOAR data products are also negatively affected by cloud cover over specific regions, which leads to poor information quality that can make this approach ineffective for PM_2.5_ retrieval. In addition, most of the studies focused only on small regions due to the difficulty of processing large-scale satellite TOAR data with high spatial and temporal resolutions. Furthermore, the discrepancy in PM_2.5_ estimations between AOD-based and TOAR-based approaches has rarely been investigated [50].

This study applied four machine-learning methods, including random forest (RF), extreme gradient boosting (XGBoost), gradient boosting regression (GBR), and support vector regression (SVR), to analyze the relationship between PM_2.5_ concentration and environmental factors in the YRD region. Spanning approximately 350,000 km^2^, the YRD is one of China’s most economically dynamic regions and has a complex topography. The study used satellite-derived TOAR and AOD data products, land-cover variables, and meteorological data as input predictors. To address the gaps in the TOAR and AOD data, a spatiotemporal convolutional approach was introduced, increasing the sample size and size of the dataset used for model training and improving model accuracy for hourly PM_2.5_ predictions. All four machine-learning models were applied to both original and gap-filled TOAR and AOD datasets to predict hourly PM_2.5_ concentrations in the YRD region. The performance of the machine-learning models was assessed using the coefficient of determination (R^2^) and root mean squared error (RMSE). The effects of sample size and spatiotemporal distribution were analyzed by comparing the performance of models based on original and gap-filled TOAR and AOD datasets. Finally, the study evaluated cardiovascular disease risks within the population residing in the YRD region.

## 2. Materials and Methods

### 2.1. Study Region

**Figure 1:**
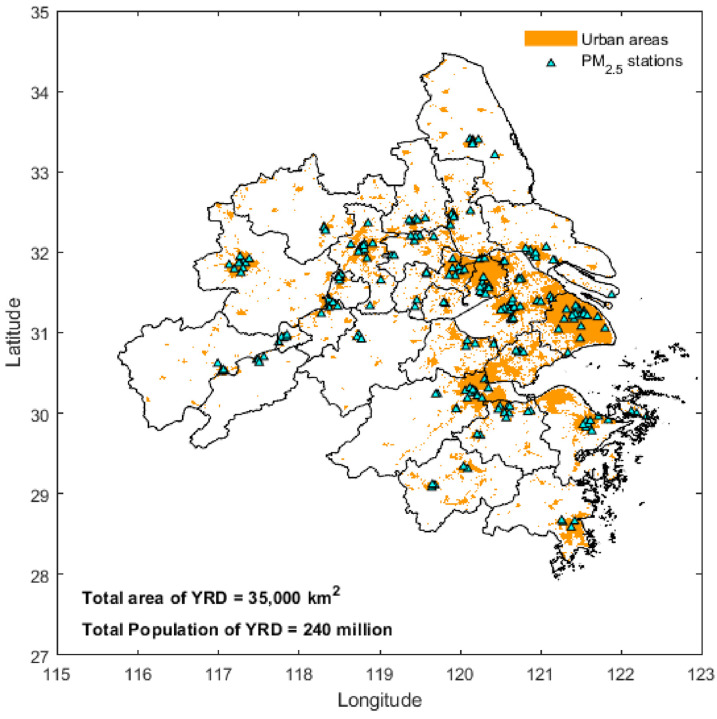
Map of study area showing the locations of air-quality-monitoring stations and major urban areas in the Yangtze River Delta (YRD) region.

In this study, we selected the YRD region (Figure 1) as the study region. The YRD, one of China’s most developed, dynamic, densely populated areas, with some of China’s most concentrated industrial development, is growing into an influential world-class metropolitan area and playing an important role in China’s economic and social development. It is also known as the economic powerhouse of modern China. As one of China’s most economically active, open, and innovative regions, the YRD region encompasses the city of Shanghai and the provinces of Jiangsu, Zhejiang, and Anhui. The YRD is one of the most populated regions in China and is home to 15% of China’s population. Moreover, the YRD region accounts for 2.2% of the national land area and contributes roughly a quarter of the national gross domestic product [34].

### 2.2. Data Sets

#### 2.2.1. Ground-Based PM_2.5_ Measurements

In this study, the hourly ground-based PM_2.5_ concentration data were obtained from 137 environmental monitoring stations in the YRD region of the China National Environmental Monitoring Center (CNEMC, http://www.cnemc.cn) in 2016. The PM_2.5_-mass concentration data provided by CNEMC were measured using the tapered element oscillating microbalance technique with an accuracy of ±1.5 μg m^−3^ to compute the hourly means. The hourly PM_2.5_ data provided by CNEMC are quality-assured and meet the national industry standard. We removed hourly measurements < 1 μg m^−3^ because that value was below the instruments’ limit of detection.

#### 2.2.2. Satellite Data Products

This study used the Level 1B TOAR and Level 2 AOD products from the Advanced Himawari Imager (AHI), which is flying onboard the Himawari-8 geostationary satellite, with a temporal resolution of 10 min and a spatial resolution of 5 km × 5 km. The TOAR values at three wavelengths 0.47 μm (blue band), 0.64 μm, and 2.30 μm (shortwave infrared) with four observation angles (sensor azimuth, sensor zenith, solar azimuth, and solar zenith) are used to obtain AOD values based on the dark target algorithm [51]. Therefore, we selected the TOAR values as the main input predictors to estimate surface PM_2.5_ concentrations. We used AOD retrievals at a wavelength of 0.50 μm with the highest confidence level from AHI Level 2 aerosol products to estimate PM_2.5_ for comparison. A cloud mask from the AHI Level 2 aerosol product was applied to retrieve cloud-free conditions. As a result, about 58% of the total satellite data acquired was lost due to the cloud-free restriction. In the NetCDF format, all these data products were obtained from the Japan Aerospace Exploration Agency P-Tree system (https://www.eorc.jaxa.jp/ptree (accessed on 15 January 2020)).

Moreover, the normalized difference vegetation index (*NDVI*) was included as an input predictor to represent land cover. *NDVI* was calculated using the following formula:

(1) $$NDVI=\frac{\rho_{0.86}-\rho_{2.26}}{\rho_{0.86}+\rho_{2.26}},$$

where *ρ*_0.86_ and *ρ*_2.26_ are the TOAR values at 0.86 and 2.26 μm, which were obtained from the AHI Level 1B reflectance products.

#### 2.2.3. Meteorological Data

Meteorological variables were obtained from the ERA-5 reanalysis data (https://cds.climate.copernicus.eu/ (accessed on 15 January 2024)). ERA-5 is the latest generation of atmospheric reanalysis data produced by the Copernicus Climate Change Service (C3S) at the European Center for Medium-Range Weather Forecast (ECMWF). ERA-5 provides hourly estimates for a wide range of land, atmosphere, and climate variables. In this study, we selected meteorological variables of high importance, including total column water (*TCW*, kg m^−2^), total column ozone (*TCO*, kg m^−2^), surface atmospheric pressure (*SP*, hPa), 10 m u-wind (*U*_10_) and v-wind (*V*_10_) components, relative humidity (*RH*, %), air temperature at 2 m height (*T*, *K*), and planetary boundary layer height (*PBLH*, m). All meteorological variables have 0.25° × 0.25° spatial and hourly temporal resolution, except *PBLH*, which is provided twice daily.

#### 2.2.4. Population Data

Population data were downloaded from the Gridded Population of the World, Version 4 [52], which is available from the Socioeconomic Data and Applications Center (https://sedac.ciesin.columbia.edu/data/collection/gpw-v4 (accessed on 13 November 2024)). We obtained the approximate populations for 2016 by linearly interpolating the population estimates from 2000 and 2010. This study employed population data with a geographical resolution of 5 km × 5 km.

### 2.3. Methods

This work included sampling, data aggregation, data preprocessing, and the implementation of four machine-learning methods to predict and validate PM_2.5_ concentrations. The ground-based PM_2.5_ hourly concentration data were collected from 137 ground monitoring stations in the YRD region. The modeling included all 137 of these sites. We aimed to identify the most effective model for predicting PM_2.5_ at specific places utilizing satellite, meteorological, and other data.

#### 2.3.1. Data Preprocessing and Matching

The hourly mean values of TOAR, AOD, observation angles, and *NDVI* were calculated for every 5 km grid in the YRD region based on AHI measurements, which were obtained during the daytime under cloud-free conditions. The hourly means for a given grid were computed by arithmetically averaging the cloud-free values, which ranged from one to six. Cloud-free grids represented 42% of the overall satellite product. All other data were integrated into the hourly AHI grids. Meteorological data were spatially interpolated to 5 km gridded values, which means that the interpolated value at a particular site is dependent on the linear interpolation of values at surrounding grid points in each dimension. Each hourly grid was allocated to its temporally nearest value. Ground-based PM_2.5_ measurements were mapped to the appropriate AHI grids.

#### 2.3.2. Imputation Method for Missing Values

In this study, we employed a flexible method for imputation of missing values based on the convolution approach. The implemented convolution kernel considers the variables’ temporal and spatial dimensions and relies on the spatial and temporal correlations in the data to predict the missing values. Previous research has shown that convolutional sliding kernels of different sizes differentially affect the number of valid pixels of variables; a large convolutional sliding kernel causes significant spatial heterogeneity in the pixel values of variables, affecting the computing performance of the imputation method [44]. Here, we set the size of the convolutional sliding kernel to 3 × 3 pixels. Filling gaps in the different selected variable values using a 3 × 3 sliding kernel involved applying a window of neighboring data points around each missing value and computing the average of non-null values within the window. The implemented method ensured the smooth filling of missing values based on the local spatiotemporal neighborhood. The convolutional kernel function can be expressed mathematically as follows:

(2) $$X_{\left(t,(i,j)\right)}=\frac{\sum_{l=-1}^{1}\sum_{m=-1}^{1}\sum_{n=-1}^{1}(X_{t+l,i+m,j+n})}{\sum_{l=-1}^{1}\sum_{m=-1}^{1}\sum_{n=-1}^{1}count|X_{t+l,i+m,j+n}|},$$

where *X*_(*t*,(*i*,*j*))_ ∊ {*AOD*, …} represents a missing data point for a particular variable *X* in temporal dimension *t* and spatial dimensions *i* and *j* (*i* = longitude, *j* = latitude). The function $\sum_{l=-1}^{1}\sum_{m=-1}^{1}\sum_{n=-1}^{1}(X_{t+l,i+m,j+n})$ returns the valid values (not null) of the variable *X* in a 3 × 3 neighborhood in both dimensions, time and space (*t*,(*i*,*j*)). The function $\sum_{l=-1}^{1}\sum_{m=-1}^{1}\sum_{n=-1}^{1}count|X_{t+l,i+m,j+n}|$ counts the number of valid values (not null) of variable *X* in the 3 × 3 neighborhood.

#### 2.3.3. Model Implementation

PM_2.5_ concentrations were estimated using two models: the TOAR-based model, which used reflection, observation angles, *NDVI*, and meteorological parameters, and the AOD-based model, which used AOD and meteorological parameters. Geographic coordinates (*Lat* (latitudes) and *Lon* (longitudes)) and dummy variables (*month*, *day*, and *hour* of observation) were also used as input predictors in these two models. The structures of these two models are indicated in the equations below:

(3) $$PM_{2.5}=f_{TOAR}\left(R1,R3,\;R6,\;Lat,\;Lon,\;Time,\;Angles,\;NDVI,\;RH,\;SP,TCW,U_{10},\;V_{10\;},T,TCO,PBLH\right),$$

(4) $$PM_{2.5}=f_{AOD}\left(AOD,\;Lat,\;Lon,\;Time,\;RH,\;SP,TCW,U_{10\;},\;V_{10\;},T,TCO,PBLH\right),$$

where *f_TOAR_* () and *f_AOD_* () represent the estimation functions for the TOAR-based and AOD-based models, respectively. The *f_TOAR_* () function uses input predictors such as top-of-atmospheric reflectance (*R1*, *R3*, and *R6*), geographical coordinates (*Lat* and *Lon*), observation time (*Time*), observation angles (*Angles*), *NDVI*, and meteorological parameters (*RH*, *SP*, *TCW*, *U*_10_, *V*_10_, *T*, *TCO*, and *PBLH*). The input predictors for the *f_AOD_* () function include *AOD*, geographical coordinates, observation time, and meteorological parameters. Both models capture the complex, nonlinear relationships between PM_2.5_ and input predictors. Therefore, machine-learning algorithms were employed to represent the estimation functions (*f_TOAR_* () and *f_AOD_* ()) due to their ability to capture nonlinear and complex relationships. Following gap-filling in the TOAR and AOD datasets, four versions of the estimation functions (*f_TOAR_*_(*original*)_ (), *f_TOAR_*_(*gap-filled*)_ (), *f_AOD_*_(*original*)_ (), and *f_AOD_*_(*gap-filled*)_) were used for model training. All four were implemented using RF, XGBoost, GBR, and SVR.

##### Random Forest (RF) Model

RF is a machine-learning model proposed by [53]; it uses an ensemble learning technique that combines multiple decision trees to make predictions. The RF model is mainly applied to classification and regression tasks by utilizing a classification and regression tree (CART) approach for learning each decision tree. The combination of different techniques, including bootstrapped sampling, random feature selection, and averaging (or majority voting) are used to create the individual decision trees in the RF; these trees aid in generating an ensemble of more generalized and precise decision trees. For regression tasks, RF uses many regressors (the multiple decision trees), and each decision tree generates a continuous real-values prediction. The final RF prediction is made by averaging the predictions of all decision trees as follows:

(5) $$\hat{Y}(x)=\frac{1}{N}\sum_{n=1}^{N}{\hat{Y}}_{n}(x),$$

where the function (${\hat{Y}}_{n}(x)$) represents the prediction from an individual decision tree for an input *x*, *N* denotes the total number of decision trees, and the function $\hat{Y}(x)$ computes the final predicted value for the input *x*. By aggregating the outputs of several decision trees, RF yields higher accuracy, reduces overfitting, and improves robustness compared to models using individual decision trees. In the training process, RF has two important parameters to tune: the number of decision trees to grow (*n_tree_*)and the number of predictors randomly sampled at each tree (*m_try_*). In this study, we utilized the grid-search approach with the 10-fold cross-validation (CV) method to optimize the RF model’s hyperparameter tuning. The best prediction accuracy was achieved when *n_tree_* and *m_try_* were set to 600 and 9 for the TOAR-based model and when *n_tree_* and *m_try_* were set to 800 and 5 for the AOD-based model.

##### Gradient-Boosting Regression (GBR) Model

GBR is another robust machine-learning algorithm widely used for regression tasks. The GBR model constructs an ensemble of decision trees (or weak learners), where each successive tree is trained to correct the errors made by the previous ones, ultimately leading to a highly efficient model [54]. The model aims to reduce the residual error from previous trees by using a gradient-descent approach to minimize a loss function before adding new trees to the ensemble. In this study, we selected the least-squares method as the loss function. Four important parameters, including the number of boosting stages (*n_estimators*), the maximum depth (*max_depth*), the fraction of samples (*subsample*), and the learning rate, were tuned. Using the grid-search approach, for the TOAR-based model, *n_estimators*, *max_depth*, *subsample*, and *learning rate* were set to 900, 7, 0.5, and 0.1. These parameters were 800, 7, 0.5, and 0.05 for the AOD-based model.

##### Extreme Gradient-Boosting (XGBoost) Regression Model

XGBoost is an advanced and optimized implementation of the GBR algorithm [55]. Similarly, to the standard gradient-boosting algorithm, it builds an ensemble of decision trees in an additive manner and implements several key optimizations to improve speed, accuracy, and scalability. This framework is widely used in various machine-learning-based real-world applications due to its high efficiency. Besides the four parameters of the GBR model, two additional parameters were also tuned for XGBoost: the subsample ratio of columns while generating individual trees (*colsample_bytree*) and the minimal sum of instance weights required in a child (*min_child_weight*). Using the grid-search approach, for the TOAR-based model, *n_estimators*, *max_depth*, *subsample*, *learning rate*, *colsample_bytree*, and *min_child_weight*, were set to 800, 9, 0.9, 0.05, 0.7, and 5, respectively. For the AOD-based model, these parameters were 900, 9, 0.7, 0.05, 0.7, and 1.

##### Support Vector Regression (SVR) Model

SVR is a machine-learning algorithm originally derived from support vector machines (SVM) that is mainly used for classification problems. SVR applies the basic idea of SVM to solve regression problems. SVR aims to find a function (hyperplane) that best fits in the feature space and optimizes the linear and non-linear relationships between the input variables and the corresponding output variables. The hyperplane is defined by a set of support vectors. These support vectors are the data points that lie within a certain margin or are close to the regression function and are vital elements in defining the optimal regression hyperplane [56]. SVR is commonly applied to predict continuous-value numerical data, such as those that appear in time-series analysis and weather forecasting. Particularly, it is trained independently based on the same training data with multiple targets and is usually used to create linear or nonlinear boundaries between input and output variables using kernel functions in forecasting tasks. This kernel function is then used to make predictions. In this study, we used the radial basis function kernel to capture the complex non-linear relationships between satellite data products, meteorological and other variables, and PM_2.5_. Based on the grid-search approach, two important parameters of SVR, namely the kernel coefficient (*gamma*) and regularization parameter (*C*), were tuned to values of 0.5 and 1 for the TOAR-based model and 0.7 and 3 for the AOD-based model.

## 3. Results

### 3.1. Performance of Different Machine-Learning Models

The RF method exhibited the highest performance for both TOAR-based and AOD-based models, whereas the SVR method achieved the lowest accuracy, as detailed in Table 1. The analysis was conducted using samples of substantial size across all datasets: the gap-filled TOAR data comprised (N = 217,125) samples, while the gap-filled AOD data included (N = 112,728) samples. Similarly, the original datasets consisted of (N = 157,898) samples for TOAR and (N = 33,663) samples for AOD, ensuring robust evaluation across all model configurations. The RF method using TOAR data arrived at the same value for the coefficient of determination (R^2^ = 0.75) for both the gap-filled and original datasets, but with a lower root mean squared error (RMSE = 18.30 μg m^−3^) value for the gap-filled data compared to the original data (RMSE = 18.85 μg m^−3^). On the other hand, the RF method with the AOD-based model yielded a higher R^2^ value (0.71) using the gap-filled AOD data than it did using the original data (0.64). It is also clear from Table 1 that all the selected machine-learning methods reached higher prediction accuracy for the TOAR-based model (highest value of R^2^ = 0.75) compared to the AOD-based model (highest value of R^2^ = 0.71).

Consistent with findings from [57], the superior performance of the TOAR-based model can be attributed to its larger sample size (N = 217,125 for TOAR vs. N = 112,728 for AOD). This aligns with machine-learning principles, which include the idea that increasing the volume of training data typically enhances model accuracy and robustness. Thus, this led to the relatively low R^2^ values of the AOD-based model. Multiple factors could have affected the AOD and TOAR data-retrieval algorithms and defined their valid dataset sizes. For example, cloud cover and other harsh atmospheric conditions could obstruct satellite AOD measurements, resulting in smaller datasets. In contrast, the TOAR data were more generalized and robust, which led to more consistent availability and a larger dataset for analysis.

**Figure 2:**
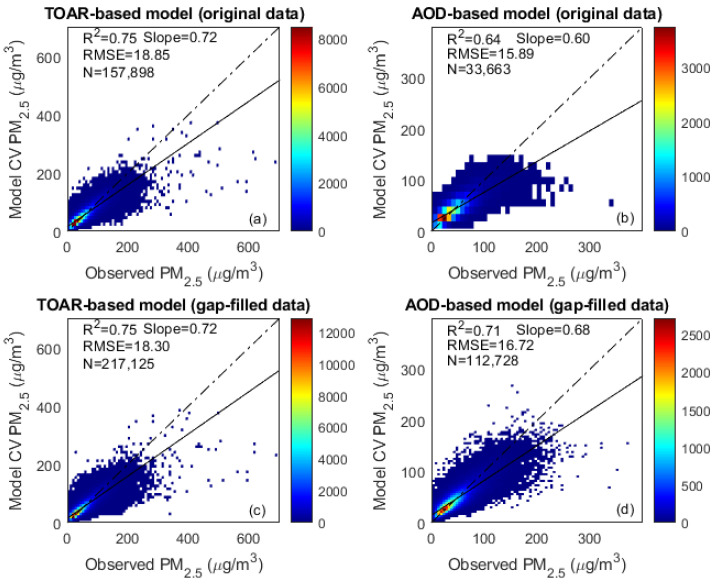
Scatter plots of cross-validation (CV) results for the (**a**) TOAR-based model; (**b**) AOD-based model; (**c**) TOAR-based model (gap-filled data); and (**d**) AOD-based model (gap-filled data) using the RF method. The solid black line is the regression line, and the dashed black line is the 1:1 reference line.

The 10-fold CV results of the RF model illustrated that the TOAR-based model outperformed the AOD-based model, with a higher value of R^2^ (0.75; Figure 2). Moreover, it was observed that both models underestimated PM_2.5_ concentrations for high values and overestimated them at low values (<50 μg m^−3^). In the YRD region, overestimations and underestimations of PM_2.5_ by TOAR and AOD models mainly arose from complex meteorological influences, inaccurate emission inventories, and limitations on satellite data. Frequent wintertime temperature inversions, boundary-layer variations, and rainfall events affect pollutant dispersion, leading to model biases. Emission changes from industrial activities and local urban effects often differ from average inventories, causing prediction mismatches. Additionally, satellite-derived TOAR and AOD data can be unreliable under cloudy or hazy conditions, introducing errors into PM_2.5_ estimates. Furthermore, machine-learning models also tend to smooth extreme pollution events (regress toward the mean), leading to underestimation at high PM_2.5_ levels and overestimation during cleaner periods (lower PM_2.5_ levels).

### 3.2. Site-Specific Performances of TOAR-Based and AOD-Based Models

**Figure 3:**
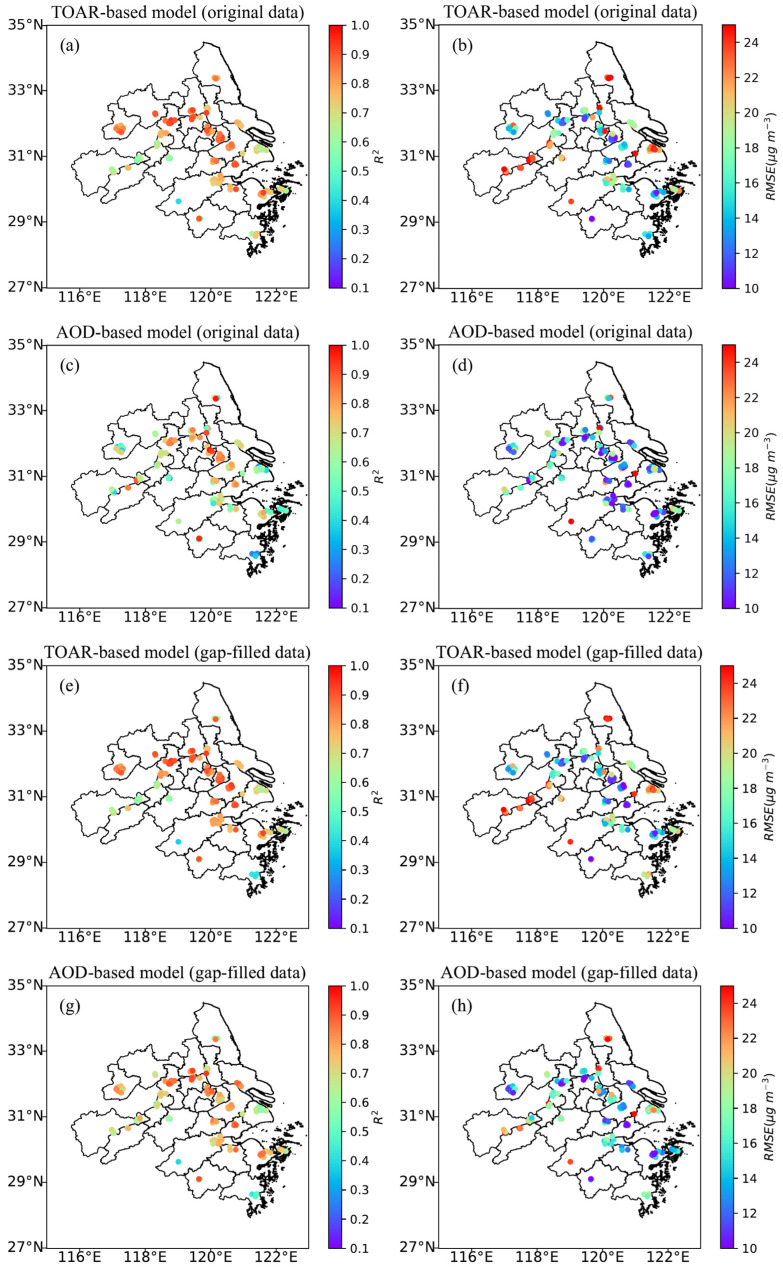
Scatter plots showing spatial variations in the coefficient of determination (R^2^) (**a**,**c**,**e**,**g**) and root mean square error (RMSE, μg m^−3^) (**b**,**d**,**f**,**h**) for each site obtained using the RF method. The (**a**,**b**,**e**,**f**) panels represent the results from TOAR-based models for original and gap-filled data, respectively. The (**c**,**d**,**g**,**h**) panels represent the results from AOD-based models for original and gap-filled data, respectively.

The site-specific performances of the TOAR-based and AOD-based models were evaluated using both gap-filled and original data with the RF algorithm (Figure 3). The TOAR-based (original data) model yielded R^2^ values ranging from 0.40 to 0.95 (Figure 3a), and the AOD-based (original data) model yielded R^2^ values ranging from 0.19 to 0.98 (Figure 3c), for all sites in the YRD region. Similarly, the TOAR-based (gap-filled data) model yielded R^2^ values ranging from 0.37 to 0.94 (Figure 3e), and the AOD-based (gap-filled data) model yielded R^2^ values ranging from 0.39 to 0.94 (Figure 3g) for all sites in the YRD region. In particular, R^2^ values were found to be close to or greater than 0.80 over the northern and eastern parts of the YRD region. In contrast, R^2^ values were observed to be less than 0.75 in the other regions of the YRD. One possible reason for lower R^2^ values in these regions could be the scarcity of sites. In contrast, the TOAR-based models using original and gap-filled data showed an opposite pattern in the spatial variation of RMSE compared to that of R^2^. Most of the sites in the northern and eastern parts of the YRD region yielded relatively lower RMSE values than the other regions of YRD (Figure 3b,f). The spatial patterns of R^2^ and RMSE for the AOD-based models, using both original and gap-filled data, were consistent with those for the TOAR-based models, as illustrated in Figure 3c,d,g,h. It can be concluded from Figure 3 that the TOAR-based model using the RF technique performed well in hourly PM_2.5_ prediction for the majority of ground-based sites in the YRD.

### 3.3. Spatiotemporal Distributions of PM_2.5_ Concentration

Because of the model’s good performance, the RF approach was used to estimate PM_2.5_ concentration levels throughout the paper using the TOAR-based (gap-filled data) and AOD-based (gap-filled data) models, unless stated otherwise.

**Figure 4:**
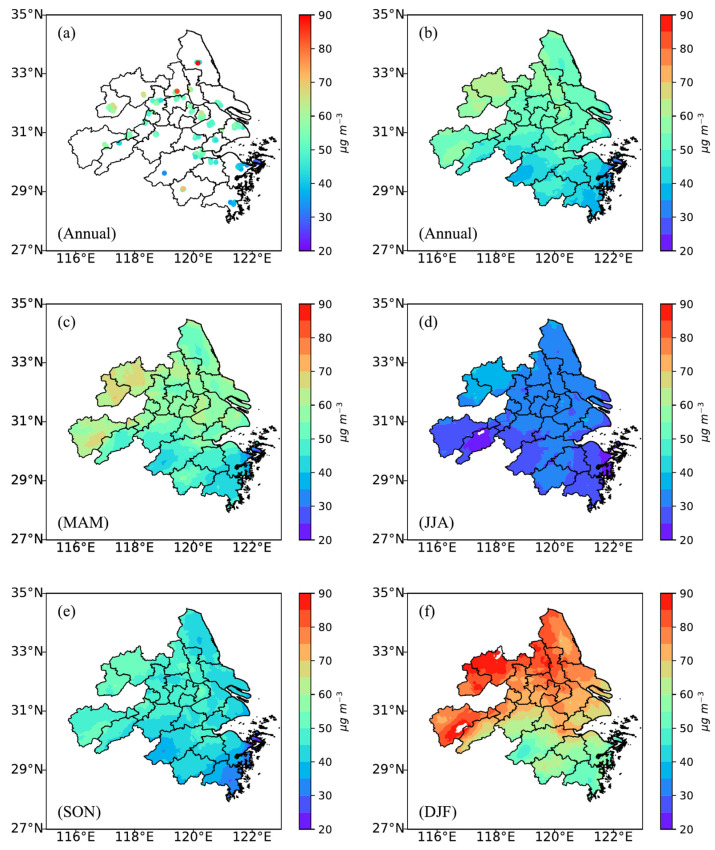
Panel (**a**) displays the spatial distribution of annual mean PM_2.5_ concentrations derived from ground-based monitoring stations across the Yangtze River Delta (YRD) region. In panel (**a**), the sites with observation periods of over half a year are plotted. Panels (**b**,**c**–**f**) depict the annual and seasonal mean PM_2.5_ concentrations from the TOAR-based model with gap-filled data and the random forest (RF) method, illustrating their spatial variability over the YRD region. MAM: March, April, May (spring); JJA: June, July, August (summer); SON: September, October, November (autumn); DJF: December, January, February (winter).

The spatial distributions of annual and seasonal mean PM_2.5_ concentration levels from the TOAR-based model over the YRD region are presented in Figure 4. Figure 4a depicts the spatial distribution of annual mean PM_2.5_ concentration levels from ground-based observations in the YRD, whereas the annual mean PM_2.5_ concentration levels estimated by the TOAR-based model are shown in Figure 4b. Both Figure 4a, and Figure 4b indicate higher PM_2.5_ concentration levels in the northern area and lower values in the southern area. It was also observed that this spatial pattern also held for seasonal mean PM_2.5_ concentration levels (Figure 4c–f). According to this study, the average annual PM_2.5_ concentration level over the YRD, estimated using the TOAR-based model, was 51.3 μg m^−3^. Winter had the highest average value, 73.3 μg m^−3^, followed by spring, i.e., 56.63 μg m^−3,^ and autumn, i.e., 45.62 μg m^−3^, and summer had the lowest value, 31.91 μg m^−3^. These findings are consistent with those from previous research [49,50].

**Figure 5:**
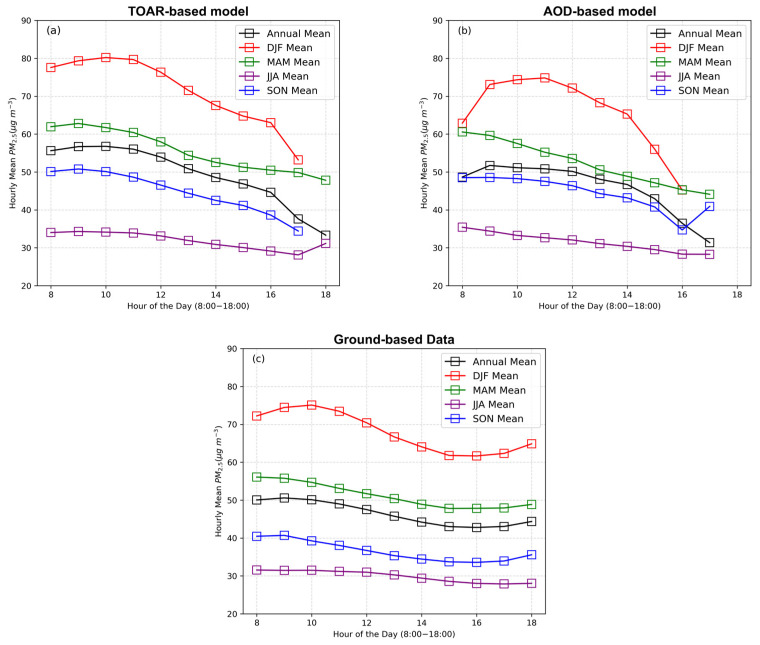
Regional hourly (local time) mean PM_2.5_ concentrations from the ground-based monitoring stations and satellite-based model predictions. The satellite-derived PM_2.5_ values are averaged over spatial grids, while ground-based values represent point measurements. Panels (**a**,**b**), respectively, represent the estimated PM_2.5_ concentrations from the TOAR-based and AOD-based models with gap-filled data and the random forest (RF) method. Panel (**c**) represents the PM_2.5_ concentrations from the ground-based-monitoring data.

Figure 5 presents the diurnal cycle (annual and seasonal) in the estimated regional mean PM_2.5_ concentration levels in the daytime (local time) based on data from the ground-based monitoring stations and based on data from the TOAR-based and AOD-based models with gap-filled data, derived using the RF method. The annual and seasonal hourly regional mean PM_2.5_ concentration levels were found to slightly increase in the early morning (8:00–10:00) and then continue to decrease toward evening in both the TOAR-based and AOD-based models (Figure 5a,b). Previous surface-measured studies in Shanghai, Hangzhou, Hefei, and Nanjing (YRD province’s capital cities) reported the same tendency in diurnal variation [50,58]. However, Figure 5b shows that the AOD-based model predicted lower values of regional hourly mean PM_2.5_ concentration levels in the winter (DJF) than did the TOAR-based model. The main possible reason for this difference in hourly mean PM_2.5_ concentration values during winter (DJF) is associated with the difference in the pixel count between TOAR and AOD data products. In winter, AOD products have a smaller sample size than TOAR products due to cloud cover, snow and ice reflection, shorter daylight hours, and high humidity. These difficulties significantly influence satellite-based AOD retrievals, which rely on clear skies and sufficient solar radiation, whereas TOAR products are more robust and less sensitive to these conditions, resulting in a greater number of valid pixels in TOAR products [46]. To further validate model performances, we compared predicted PM_2.5_ concentrations with hourly ground-based measurements (Figure 5). The satellite-based model slightly overestimates PM_2.5_ levels during the morning hours and underestimates them in the evening. These differences can be attributed to two main factors. First, the model predictions are grid-based averages (e.g., 5 km × 5 km), while ground stations capture point-level data, which may include sharper local pollution peaks. Second, during evening hours (e.g., 18:00 local time), satellite-derived predictions are often unavailable due to limitations in AOD and TOAR data retrieval that are caused by reduced solar illumination, increased cloud cover, and retrieval-quality constraints. As a result, evening comparisons rely only on ground-based data, which may capture elevated PM_2.5_ levels not reflected in the outputs from the satellite-based model.

### 3.4. PM_2.5_ and Cardiovascular Disease

**Figure 6:**
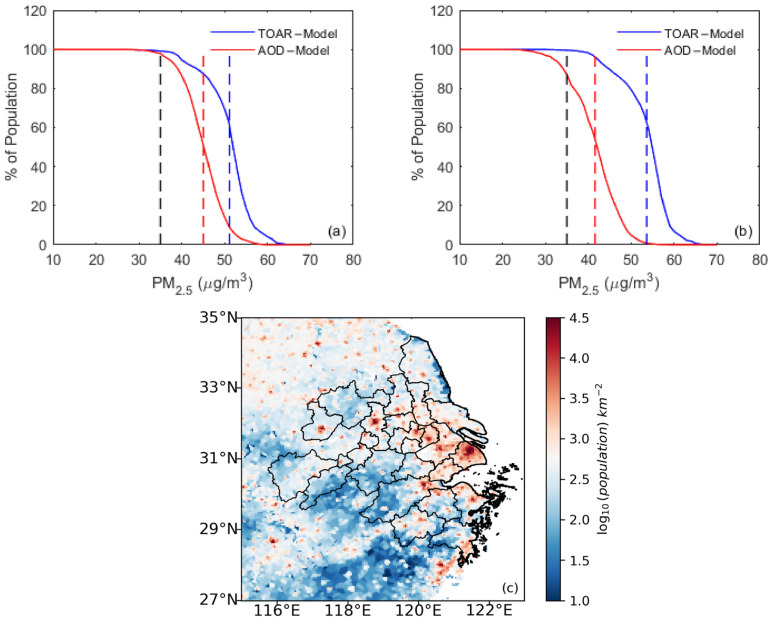
The cumulative distribution function of annual mean PM_2.5_ in YRD. (**a**) Cumulative distribution of annual means for the TOAR-based and AOD-based models, as calculated using the gap-filled dataset; (**b**) cumulative distribution of annual means for the TOAR-based and AOD-based model, as calculated using the original dataset; (**c**) population density. The solid blue and red lines represent the PM_2.5_ estimates obtained using the TOAR-based and AOD-based models, respectively. The dashed black line represents the national annual air-quality standard (35 μg m^–3^). The blue and red dashed lines denote the population-weighted means of PM_2.5_ concentrations obtained using the TOAR-based and AOD-based models, respectively.

To evaluate the health impacts of PM_2.5_ exposure with respect to cardiovascular disease in the population of the YRD region, we compared regional PM_2.5_-exposure estimates derived from TOAR-based and AOD-based models using both the gap-filled and the original datasets. Figure 6 illustrates the cumulative distribution of annual mean PM_2.5_ concentrations (Figure 6a,b) and population density (Figure 6c) across the YRD region. The statistics in Figure 6a,b were computed by aggregating the population in each pixel with an annual mean PM_2.5_ concentration equal to or exceeding a specified threshold. The population-weighted mean PM_2.5_ concentrations for the TOAR-based and AOD-based models, as estimated using the gap-filled dataset, were 51 μg m^−3^ and 45 μg m^−3^, respectively (Figure 6a). For the original dataset, the corresponding values were 54 μg m^−3^ and 42 μg m^−3^ (Figure 6b). These results indicate that nearly the entire population in the YRD resided in non-attainment regions (non-attainment regions are defined as areas where annual mean PM_2.5_ concentrations exceed China’s national air-quality standard of 35 μg m^−3^), as illustrated by the blue and red lines in Figure 6a,b. However, a small proportion of the population (16%) lived in attainment regions (attainment regions are defined as areas where annual mean PM_2.5_ concentrations fall below China’s national air quality standard of 35 μg m^−3^) under the AOD-based model using the original dataset, as shown by the red lines in Figure 6b.

According to the Chinese air-quality standard, the annual average PM_2.5_ concentration limit is 35 μg m^–3^, which is greater than the maximum annual average limit set by the World Health Organization (WHO) (i.e., 15 μg m^−3^). In comparison, the United States recommends annual average PM_2.5_ concentrations of less than or equal to 12 μg m^−3^, while European standards permit an annual average of less than or equal to 20 μg m^−3^. However, significant PM_2.5_-associated risk factors for cardiovascular health have been observed at concentrations below these levels. For instance, chronic exposure to PM_2.5_ pollution has been reported to increase major risk factors for cardiovascular disease in Americans by eight to twenty percent or more for every 10 μg m^−3^ rise in PM_2.5_ concentrations [3]. This study highlights that the majority of the population in the YRD region is at risk of developing cardiovascular diseases due to prolonged PM_2.5_ exposure. Consequently, it is essential to implement measures to reduce PM_2.5_ levels and mitigate their adverse health effects in the region.

### 3.5. The Impact of Pixel-Count (Sampling) Differences on TOAR-Based and AOD-Based Models

**Figure 7:**
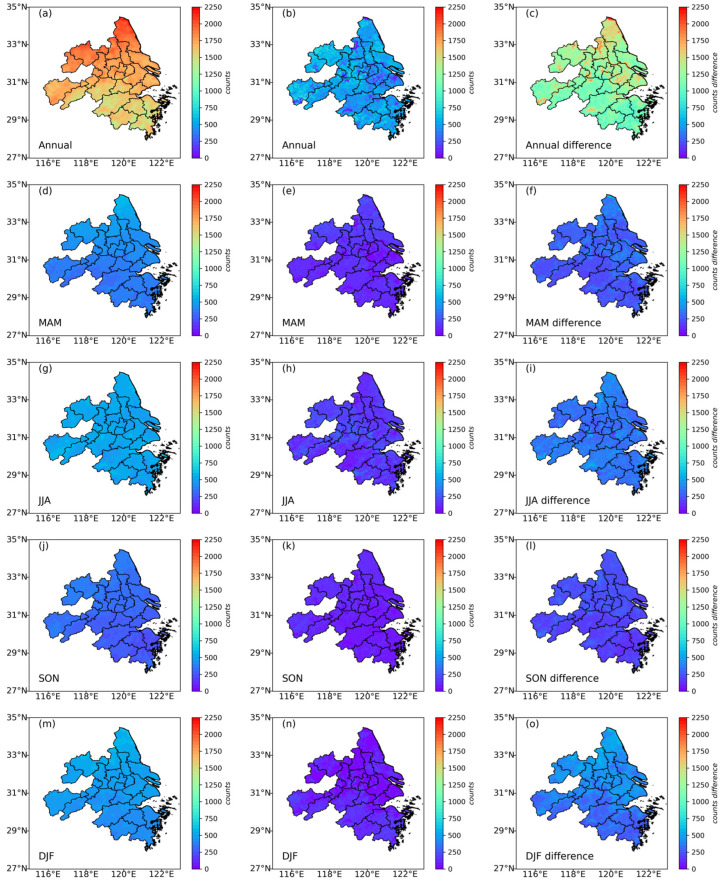
Spatial distribution of annual and seasonal pixel counts for different models. The lefthand panels (**a**,**d**,**g**,**j**,**m**) show the spatial distributions of annual and seasonal pixel counts for the TOAR-based (gap-filled data) model. The middle panels (**b**,**e**,**h**,**k**,**n**) show the spatial distributions of annual and seasonal pixel counts for the AOD-based (original data) model. The righthand panels (**c**,**f**,**i**,**l**,**o**) show the spatial distributions of annual and seasonal differences in the pixel counts between the TOAR-based (gap-filled data) and AOD-based (original data) models. MAM: March, April, May (spring); JJA: June, July, August (summer); SON: September, October, November (autumn); DJF: December, January, February (winter).

The regional spatial distributions of pixel-count (sample) differences for the TOAR-based and AOD-based models are depicted in Figure 7. Satellite TOAR and AOD data products are composed of discrete pixels, each representing an individual grid cell (e.g., 5 km × 5 km for AHI/Himawari-8) for which atmospheric reflectance or aerosol optical depth is retrieved. In practice, having more TOAR and AOD pixels means greater spatial coverage and data density, allowing for finer, more detailed monitoring of air-pollution patterns over a region. This improves the accuracy and reliability of air-quality assessments and models, especially in heterogeneous or urban environments like YRD.

A comparison of the annual and seasonal differences in pixel counts (right panels in Figure 7) between the TOAR-based (gap-filled data) and AOD-based (original data) models revealed that the southern part of the YRD region exhibits relatively smaller differences. This is likely due to the higher proportion of dark pixels in this area, which enhances the accuracy of AOD retrieval, as the area has a high proportion of forest [59]. The annual differences in the number of pixels between the TOAR-based (gap-filled data) and AOD-based (original data) models (Figure 7b) were mostly caused by the winter contribution (Figure 7o). This sampling gap in winter also contributed to significant differences in the magnitude of PM_2.5_ estimations between the two models. Referring to previous studies, the number of valid pixels in winter is significantly lower for AOD than for TOAR due to challenges such as cloud cover, snow and ice reflection, shorter daylight hours, and high humidity. These conditions adversely affect satellite-based AOD retrievals, which depend on clear skies and adequate solar radiation. In contrast, TOAR retrieval algorithms are more robust and less sensitive to such conditions, resulting in a greater number of valid pixels [36,60]. Moreover, the numbers of pixels for both TOAR and AOD observations were notably low in the autumn. This is mainly due to aerosols being misclassified as clouds underneath intense pollution caused by heavy crop-waste burning in SON in the YRD region. In terms of spatial distribution and seasonal variation, the TOAR-based model demonstrated greater reliability (left panels in Figure 7).

**Figure 8:**
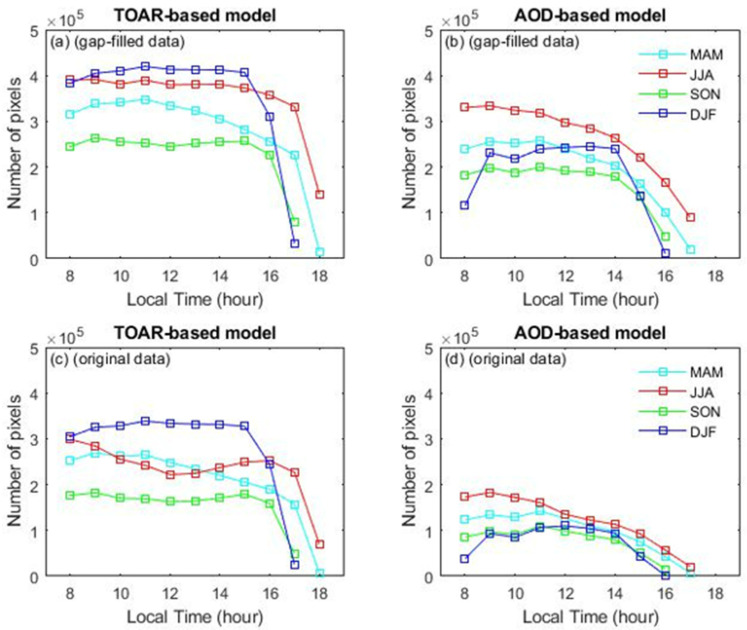
Number of pixels recorded each hour in the daytime over the YRD region. Panels (**a**,**b**) present results from the TOAR-based (gap-filled data) and AOD-based (gap-filled data) models, respectively. Panels (**c**,**d**) illustrate results from the TOAR-based (original data) and AOD-based (original data) models, respectively. MAM: March, April, May (spring); JJA: June, July, August (summer); SON: September, October, November (autumn); DJF: December, January, February (winter).

Furthermore, we examined the number of pixels per hour obtained from the TOAR-based and AOD-based models using original and gap-filled datasets (Figure 8). The number of hourly pixels during daytime across all models exhibited a consistent trend: a slight increase in the early morning (8:00–10:00), followed by a gradual decline toward the evening. However, the TOAR-based model with gap-filled data consistently had more pixels than any of the other models, regardless of the hour or season. The most significant difference was observed between the TOAR-based (gap-filled data) and AOD-based (original data) models, with the former having substantially more pixels than the latter. Specifically, the greatest difference occurred during the winter. The difference in the number of pixels observed between the TOAR-based and AOD-based models is primarily due to seasonal variations and data-quality issues. In winter, cloud cover and lower sunlight significantly reduce the availability of AOD data, especially during hours of high aerosol concentration, leading to there being fewer usable pixels for the AOD-based model. On the other hand, gap-filling techniques in the TOAR-based and AOD-based models help compensate for missing data, ensuring more consistent pixel coverage, even in cloudy conditions. This is especially noticeable in winter, when atmospheric conditions such as increased pollution and persistent clouds further increase data gaps in the AOD-based model.

**Figure 9:**
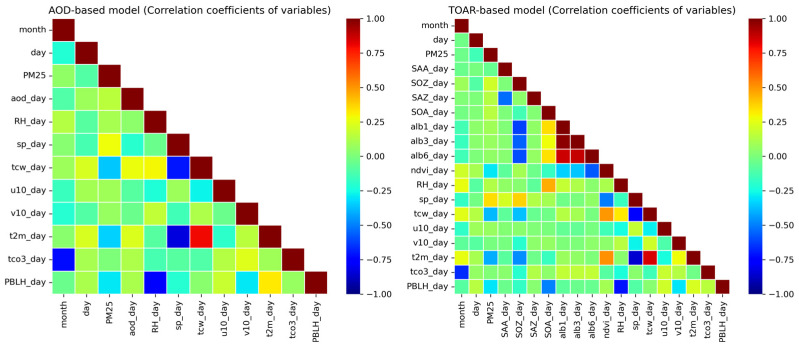
Correlation coefficients between PM_2.5_ concentrations and predictor variables in the study area.

Figure 9 illustrates the correlations between the variables utilized in this study, which were evaluated to determine their significance. This study observed that in the YRD region, the correlation between PM_2.5_ and AOD observations was weaker than expected, especially compared to findings from Beijing in previous research by Jia et al. [38]. This discrepancy can be attributed to several factors specific to the YRD’s geography and climate. The region is characterized by high-altitude mountains, densely populated urban areas, and significant land-sea contrasts, which create complex aerosol and pollution dynamics. Moreover, the humid subtropical climate of the YRD, which is marked by high humidity and frequent cloud cover, significantly hampers the accuracy of satellite TOAR and AOD retrievals, reducing the quality of satellite data. This may be the primary reason why the correlation between AOD and PM_2.5_ is not as strong as has been reported in regions like Beijing, where the topography and weather conditions are different. In contrast, meteorological variables such as *v10_day* (*V*_10_), *u10_day* (*U*_10_), and *sp_day* (*SP*) showed the highest positive values for correlation with PM_2.5_ levels. On the other hand, variables like *PBLH_day* (*PBLH*), *tcw_day* (*TCW*), and *t2m_day* (*T*) exhibited strongly negative correlations with PM_2.5_. Interestingly, *tcw_day* (*TCW*) and *t2m_day* (*T*) showed highly positive correlations with AOD, indicating that while meteorological factors are influential in predicting PM_2.5_ levels, they also play a role in AOD variations. Overall, the study highlights that meteorological factors such as wind patterns, temperature, and humidity have a stronger influence on PM_2.5_ predictions than on satellite-derived AOD in the YRD, especially in the presence of complex environmental conditions.

## 4. Discussion and Conclusions

In this study, four machine-learning models, RF, XGBoost, GBR, and SVR, were employed to establish the relationship between satellite TOAR and AOD data products and PM_2.5_ concentrations. To validate the models’ performances, ground-based data were collected from 137 climate-monitoring sites across the YRD region (Figure 1). To ensure continuous spatiotemporal coverage of satellite data products and mitigate the impact of harsh weather conditions, a flexible method for imputation of missing values based on a spatiotemporal convolution approach was employed to fill gaps in satellite TOAR and AOD observations using a 3 × 3 sliding kernel. We also estimated the PM_2.5_ concentrations by applying machine-learning methods to the original TOAR and AOD data to validate the accuracy of the gap-filled data results.

Based on the different performances of the machine-learning models, the RF model was finally selected to estimate the PM_2.5_ concentrations across the YRD region in 2016 using both gap-filled and original datasets (Table 1). Notably, the TOAR-based model with gap-filled data achieved the highest accuracy, with R^2^ = 0.75 and RMSE = 18.30 μg m^–3^, among all the models (see Table 1). The largest performance difference was observed between the TOAR-based (gap-filled data) and AOD-based (original data) models. However, the TOAR-based model with gap-filled data (R^2^ = 0.75 and RMSE = 18.30 μg m^–3^) showed only marginal improvements over the TOAR-based model with original data (R^2^ = 0.75 and RMSE = 18.75 μg m^–3^), with a slight reduction in RMSE (difference = 0.45 μg m^–3^). One possible reason for the better performance of the TOAR-based model could be the difference in sample sizes, as the number of samples can significantly influence the performance of machine-learning algorithms [57]. Note that the sample size of the AOD-based (original data) model was about one-sixth that of the TOAR-based (gap-filled data) model. This led to the R^2^ values of the AOD-based model being relatively low. Multiple factors may have affected the TOAR and AOD data-retrieval algorithms and defined their valid dataset size. For example, cloud cover and other harsh atmospheric conditions could obstruct satellite AOD measurements, resulting in smaller datasets. In contrast, the TOAR data were more generalized and robust, which led to more consistent availability and a larger dataset for analysis.

Moreover, several factors influence the relationship between satellite TOAR and AOD data products and surface PM_2.5_. The vertical distribution of aerosols is the most critical one and can be obtained by lidar retrievals. Space-based lidar, such as Cloud-Aerosol Lidar with Orthogonal Polarization (CALIOP), has extremely limited horizontal coverage due to its narrow swath and 16-day repeat cycle. Although studies have shown CALIOP’s potential for estimating PM_2.5_ within a few hundred kilometers of observation sites in the United States [61]. Its applicability in the YRD region is less certain due to the region’s higher PM_2.5_ pollution intensity and lower spatial coverage of observation sites. Further research is needed to determine whether CALIOP measurements can accurately estimate PM_2.5_ across the YRD region. Furthermore, we extended the study area to include the YRD and nearby cities, following Bi et al.’s approach [41]. Incorporating data from additional ground-based sites resulted in minimal changes to the model’s performance, indicating that the existing ground-based PM_2.5_ observation sites in the YRD region in 2016 were sufficient to effectively monitor PM_2.5_ concentrations.

The model’s prediction accuracies exhibited clear seasonal variations. For instance, the TOAR-based model (using gap-filled data and the RF method) yielded the greatest cross-validated R^2^ value in winter (0.74), followed by fall (0.70), spring (0.61), and summer (0.54). Consistently, winter was associated with the highest RMSE value (22.59 μg m^–3^), whereas summer had the lowest (11.65 μg m^–3^). Notably, the models tended to underestimate PM_2.5_ concentrations at higher levels, particularly during winter, which likely contributed to the elevated RMSE values during this season. Wintertime pollution events are often more severe and are influenced by complex meteorological conditions, such as temperature inversions, frozen air masses, and enhanced formation of secondary aerosols, and they are thus more difficult for models to capture accurately. Moreover, the training dataset contained relatively fewer high-concentration samples, potentially biasing the models toward better performance at moderate pollution levels and underestimating extreme events. These factors together explain the observed seasonal differences in model accuracy and error metrics. The seasonal variability is largely attributed to the peak PM_2.5_ concentrations during winter, which resulted in worse RMSE performance in winter compared to other seasons (see Figure 4).

Previous studies have predominantly used AOD as the primary predictor for estimating PM_2.5_ concentrations [39]. However, our findings reveal that both the TOAR and AOD-based models overestimate PM_2.5_ at lower concentration levels and underestimate it at higher levels in the YRD region (see Figure 2a,d). These biases are mainly attributed to complex meteorological conditions, inaccuracies in emission inventories, and limitations of satellite data. Frequent wintertime temperature inversions and data gaps under cloudy conditions further introduce uncertainties. Additionally, machine-learning models often smooth extreme events, leading to underestimation of high PM_2.5_ concentrations and overestimation of low concentrations during cleaner periods. Despite these challenges, the TOAR-based model with gap-filled data, benefiting from a larger sample size, demonstrated better prediction performance. However, it still faces limitations under cloudy conditions and during nighttime. Model predictability could be further improved through the use of datasets with higher temporal and spatial resolution and the implementation of more robust data-gap-filling approaches.

Both models exhibited a diurnal cycle in regional mean PM_2.5_ in YRD during the daytime, with concentrations slightly increasing in the early morning (8:00–10:00) and gradually decreasing in the evening (see Figure 5). The TOAR-based model accurately captured the spatial distribution of annual mean PM_2.5_, with higher levels in the north and lower levels in the south. Based on the TOAR-based (gap-filled data) model, the regional population-weighted annual mean PM_2.5_ concentration was 51 μg m^−3^. This indicates that almost all populations in the YRD in 2016 resided in non-attainment areas, which puts those people at risk for several cardiovascular diseases (see Figure 6).

A limitation of this study is the unavailability of real-time, region-specific data on cardiovascular disease in the YRD region, which prevented a direct validation of the health impacts of PM_2.5_ exposure. In future work, we aim to incorporate actual health-outcome data through national-level studies or collaborations to more robustly substantiate the association between air pollution and cardiovascular disease risks. Additionally, we will focus on enhancing the model’s predictability by employing datasets with higher temporal and spatial resolution (e.g., <1 km), expanding the study area, and integrating additional data sources. We also plan to assess the health impacts of PM_2.5_ exposure on a national scale. Furthermore, deep-learning approaches will be explored to improve the gap-filling of TOAR and AOD data, and their effects on model performance will be systematically evaluated.

## Figures and Tables

**Table 1:** Performance of different machine-learning algorithms.

Machine-Learning Algorithms	TOAR-Based Model			AOD-Based Model		
	R^2^	RMSE (μg m^−3^)	Slope	R^2^	RMSE (μg m^−3^)	Slope
RF (gap-filled data)	0.75	18.30	0.72	0.71	16.72	0.68
RF (original data)	0.75	18.85	0.72	0.64	15.89	0.60
GBR (gap-filled data)	0.71	20.42	0.73	0.67	17.79	0.68
XGBoost (gap-filled data)	0.73	19.44	0.71	0.69	17.45	0.66
SVR (gap-filled data)	0.69	20.95	0.68	0.65	18.85	0.65

R^2^: coefficient of determination; RMSE: root mean square error; RF: random forest; GBR: gradient-boosting regression; XGBoost: extreme gradient boosting; SVR: support vector regression.

## Data Availability

Data used in the reported studies were obtained from websites, as indicated in the text.
